# The Relationship Between Religious Coping and Internalized Stigma Among Patients With Bipolar Disorder

**DOI:** 10.7759/cureus.43511

**Published:** 2023-08-15

**Authors:** Ayşe Erdoğan Kaya, Beyza Erdogan Akturk

**Affiliations:** 1 Psychiatry, Hitit University Çorum Erol Olçok Training and Research Hospital, Çorum, TUR; 2 Psychiatry, Tarsus State Hospital, Mersin, TUR

**Keywords:** coping, stigma, religious coping, internalized stigma, bipolar disorder

## Abstract

Background: Stigmatization is a situation that results from the negative perspective of society toward individuals with certain mental and physical illnesses and has negative effects. It has been observed that there are not enough studies in the literature investigating the attitudes of individuals with mental illness to cope with lifelong difficulties such as stigma and especially their religious coping attitudes. However, there are many clinical studies on general psychology and religious coping with varying results. Our aim in this research is to reveal the association between religious coping and internal stigma among bipolar disorder patients.

Methods: The religious coping scale and the Internalized Stigma in Mental Illness (ISMI) scale were administered to 79 patients with bipolar disorder. The obtained data were analyzed using IBM SPSS Statistics for Windows, Version 22 (Released 2013; IBM Corp., Armonk, New York, United States).

Results: Forty-two (53.1%) patients were female and 37 (46.9%) were male, the mean age was 43.41±12.57, and the mean follow-up period was 11.95±9.15 years. A positive correlation was found between negative religious coping and discrimination experience, alienation, and social withdrawal in bipolar disorder patients. A significant negative correlation was found between discrimination experience, alienation and social withdrawal, and positive religious coping.

Conclusions: The correlation of religious coping attitudes with discrimination experience, alienation, and social withdrawal makes us think that religious coping methods may be one of the issues to be considered when dealing with self-stigma in bipolar disorder patients. In addition, the relationship between religious coping and self-stigmatization in mental illnesses can add a new dimension to psychosocial approaches. It would be beneficial for authors interested in religion and social psychology to focus on more extensive research on this subject.

## Introduction

Bipolar disorder is a chronic mental illness accompanied by depression, mania, or mixed episodes, in which functionality often improves significantly between episodes [1]. One of the problems faced by individuals with bipolar disorder and those with other severe mental illnesses in society is stigmatization [2]. Stigmatization, which is associated with situations such as contempt and exclusion of individuals with mental problems by society, results in many negative situations [3]. Internalized stigma is a psychological state that emerges with situations such as shame, secrecy, and social withdrawal that arise as a result of the acceptance of the negative stereotypes of the people in the environment by the individual himself [4]. Especially the acceptance of stigma by individuals with mental illness by society leads to internalized stigma, which leads to a decrease in self-confidence and quality of life [4]. Internalized stigma, known for its negative effects on mental illnesses, is a topic that has been researched many times both in our country and in the world but is still on the agenda [5-7].

It is known that individuals with mental illness use various coping strategies when faced with difficult life events, and one of them is religious coping strategies [8,9]. The religious belief system is an important issue for many people as it provides relief from a religious point of view while struggling with problems [10]. Positive religious coping is manifested by religious thoughts and behaviors that emerge with a strong relationship with God and focusing on his love, while negative religious coping is an attitude that includes thoughts of abandonment and punishment by God, and both positive religious coping and negative religious coping are known to be associated with mental health outcomes [11].

There are many studies in the literature investigating spirituality and religious coping in bipolar disorder patients. But in general, in-depth knowledge and clinical research in the literature regarding the relationship between internalized stigma and religious coping have not been identified, and research has been limited to only a one-sided examination of religious coping or only internalized stigma [12,13]. According to a review article, it was emphasized that religion and spirituality are important in terms of the psycho-social model in bipolar disorder, but evidence and cause-effect relationships need to be developed [12]. Another study found that religiosity and positive religious coping were associated with higher quality of life and fewer depressive mood symptoms in bipolar disorder [13]. Although religious coping is a subject that has been studied before in bipolar disorder, literature data on its relationship with internalized stigma are insufficient. Our aim in this study is to reveal whether internalized stigma is associated with positive and negative religious coping in bipolar disorder because the number of clinical studies examining these two together is insufficient in the literature. Our hypothesis is that with the increase of internalized stigma, negative religious coping attitudes with negative God perception will also increase.

## Materials and methods

This study was planned as a cross-sectional study and carried out with the permission of Hitit University Faculty of Medicine Clinical Researches Ethics Committee (Date: 2023, Decision No:45). All procedures were carried out in accordance with the ethical rules and the principles of the Declaration of Helsinki. All individuals included in the study signed the informed voluntary consent form. Seventy-nine bipolar disorder patients who applied to the psychiatry outpatient clinic and met the inclusion criteria were included in the study. Inclusion criteria for the study, according to DSM-V, meeting the diagnostic criteria for bipolar disorder were patients between 18 and 65 years, being literate, being in the euthymic phase, and volunteering to participate in the study. Exclusion criteria are having alcohol substance use disorder, having an accompanying mental or neurological disease, being under the age of 18, being over 65 years of age, having acute exacerbation symptoms of bipolar disorder and illiteracy, and not agreeing to participate in the study. The Internalized Stigma in Mental Illness (ISMI) scale and the religious coping scale were applied to the patients included in the study, and the results were analyzed with IBM SPSS Statistics for Windows, Version 22 (Released 2013; IBM Corp., Armonk, New York, United States). Descriptive statistics, Kolmogorov-Smirnov, and Shapiro-Wilk normal distribution tests were used. Due to the sample size of the study, non-parametric tests were preferred. The Mann-Whitney U test was used to compare means, the Pearson chi-square test and Fisher's exact test were used to evaluate categorical variables, and Spearman correlation analysis was used for correlation evaluation. p value<0.05 was considered significant.

The ISMI scale

ISMI, developed by Ritsher et al., is a 29-item self-report scale that evaluates internalized stigma [14]. The Turkish adaptation study of the scale was carried out by Ersoy and Varan on psychiatric patients admitted to the outpatient unit [15]. The scale has five subgroups: alienation, stereotype endorsement, discrimination experience, social withdrawal, and stigma resistance. Alienation subscale measures the subjective experience of not being a full member of society, stereotype endorsement measures the extent to which society accepts common stereotypes about individuals, discrimination experience measures perceptions of how they are treated by others, social withdrawal measures socially withdrawn aspects, while stigma resistance measures the degree to which a person resists internalized stigma. The score to be obtained from the scale varies between 29 and 116. The Cronbach alpha internal consistency of the scale was reported as 0.93. An increase in the total score means that the person's internalized stigma is more severe in the negative direction [14,15].

Religious coping scale

This self-report scale shows the religious coping activities used by individuals in challenging situations and the frequency of their use. It was developed in 2008 by Abu-Raiya et al. [16]. A Turkish adaptation study was carried out by Ekşi and Sayın in 2006 [17]. Psychometric studies of the Turkish form have shown that the scale has the same structure as the original form. The Cronbach's alpha internal consistency coefficient of the scale was 0.91 and 0.86 for the positive and negative religious coping subscales, respectively. The scores that can be obtained from the positive and negative religious coping subscales range from 7 to 28 and 3 to 12, respectively. Low scores from the scale prepared in a four-point Likert-type format indicate that religious coping style is used less, and high scores indicate that it is used more [17].

## Results

Seventy-nine patients who met the inclusion criteria were included in our study. Forty-two of the patients were female and 37 were male, the mean age was 43.41±12.57, and the mean follow-up period was 11.95±9.15 years. Thirty-eight patients were single and 41 of them were married. While 12 patients were working in a job, 28 patients were not working and 39 patients were retired.

The ISMI total score average was 61.94±19.22. The sub-dimension averages of the scale were as follows: alienation 14.10±6.86, stereotype endorsement 14.49±6.38, discrimination experience 12.28±5.71, stigma resistance 8.08±3.17, and social withdrawal 12.99±5.74. In the results of the religious coping scale, the mean positive religious coping score was 14.72±5.28, and the mean negative religious coping score was 8.035±3.46.

While the age of the patients showed a normal distribution, other parameters did not show a normal distribution. The demographic data and scale results of the patients by gender are given in Table 1.

**Table 1:** Comparison of demographic data and scale scores by gender Mann-Whitney U and Pearson chi-square tests were used. p<0.05 was considered significant. ISMI: Internalized Stigma in Mental Illness

	Female (n:42)	Male (n:37)	p-value
Age	43.45±11.83	43.35±13.52	0.875
Marital status	Single: 35.7% (n:15)	Single: 62.2% (n:23)	0.019
	Married: 64.3% (n:27)	Married: 37.8% (n:14)	
Working status	Working: 4.8% (n:2)	Working: 27.0% (n:10)	0.002
	Non-working: 50.0% (n:21)	Non-working: 18.9% (n:7)	
	Retired: 45.2% (n:19)	Retired: 54.1% (n:20)	
Illness follow-up period	11.64±8.30	12.30±10.13	0.969
Alienation	13.24±6.95	15.08±6.73	0.106
Stereotype endorsement	13.31±5.98	15.84±6.62	0.133
Discrimination experience	11.52±5.58	13.13±5.80	0.179
Stigma resistance	8.02±3.02	8.14±3.37	0.929
Social withdrawal	11.55±5.24	14.62±5.91	0.019
ISMI total score	57.64±18.37	66.81±19.23	0.030
Positive religious coping	16.07±5.43	13.19±4.71	0.017
Negative religious coping	7.31±3.48	8.84±3.30	0.076

The correlations between the age of the patients and the scores of the religious coping scale and internalized stigma were evaluated; a statistically significant negative correlation was found between age and social withdrawal (r: -0.224 p=0.047) and stereotype endorsement (r: -0.273 p=0.015), but no significant correlation was found with other scale scores.

A significant and negative correlation was detected between positive religious coping and internalized stigma total score, social withdrawal, discrimination experience, and alienation sub-dimension scores. A significant and positive correlation was found between negative religious coping and internalized stigma total score, social withdrawal, discrimination experience, and alienation sub-dimension scores. The correlation analysis results between religious coping and internalized stigma sub-dimensions are given in Table 2. In addition, a statistically significant and strong negative correlation was found between negative religious coping and positive religious coping (r: -0.810 p<0.001).

**Table 2:** Correlations between religious coping and internalized stigma sub-dimensions Spearman's correlation test was applied. p<0.05 was considered significant. ISMI: Internalized Stigma in Mental Illness

	Positive religious coping	Negative religious coping
Alienation	r: -0.770 p < 0.001	r: 0.754 p < 0.001
Stereotype endorsement	r: -0.205 p = 0.069	r: 0,113 p = 0.322
Discrimination experience	r: -0.705 p < 0.001	r: 0.729 p < 0.001
Stigma resistance	r: -0.141 p = 0.216	r: -0.094 p = 0.408
Social withdrawal	r: -0.713 p < 0.001	r: 0.784 p < 0.001
ISMI total score	r: -0.768 p < 0.001	r: 0.701 p < 0.001

## Discussion

According to the main findings of our study, a significant and negative correlation was detected between positive religious coping and internalized stigma total score, social withdrawal, discrimination experience, and alienation sub-dimension scores. A significant and positive correlation was found between negative religious coping and internalized stigma total score, social withdrawal, discrimination experience, and alienation sub-dimension scores. Additionally, the correlations between the age of the patients and the scores of the religious coping scale and internalized stigma were evaluated; a statistically significant negative correlation was found between age and social withdrawal and stereotype endorsement, but no significant correlation was found with other scale scores.

In many studies investigating stigma and related factors in severe mental illnesses, it has been determined that different conditions such as quality of life, adherence to treatment, self-esteem, and depressive symptoms are associated with internalized stigma [18-20]. In our study, it was tried to reveal the association between self-stigma and religious coping, which is less researched in the literature.

According to our results, positive and negative religious coping scores of the patients were negatively correlated. It is expected that individuals with a positive perception of religion and God will have less negative feelings and thoughts toward God and have a more hopeful perspective. As a result of the relevant literature review, it was seen that there were similar results in previous studies [21].

The significant and negative relationship between positive religious coping and discrimination experience, social withdrawal, and alienation, which is one of the main findings of our research, may be due to the spiritually relaxing effects of a positive perception of God and focusing on God's mercy. This may be related to the contribution of positive religious coping to mental well-being, as positive religious coping has been associated with fewer depressive symptoms, better physical functioning, and higher quality of life in previous studies [22]. Although there is not enough literature investigating internalized stigmatization and religious coping in individuals with mental illnesses, our findings suggest that positive religious coping may lead to lower self-stigmatization in individuals with bipolar disorder. However, it is not known whether this effect is a direct effect or an indirect effect.

The fact that the stigma resistance sub-dimension is not significantly associated with positive and negative religious coping can be explained by the weak relationship between stigma resistance and religious coping. According to a study on bipolar disorder patients investigating the relationship between quality of life and internalized stigma; while stereotype endorsement, discrimination experience, social withdrawal, and alienation sub-dimensions were associated with quality of life, the correlation between stigma resistance sub-dimension and quality of life was rather weak [2]. According to the findings of our study, the lack of a significant relationship between religious coping and resistance to stigma sub-dimension may be related to the fact that this sub-dimension may affect the quality of life less than other factors.

According to the other results of our study is that the internalized stigma total score and social withdrawal scores were significantly higher in male patients than in female patients. Alienation, stereotype endorsement, discrimination experience, and stigma resistance subscale scores did not differ significantly between genders. When the literature data investigating internalized stigma in mental illnesses are examined, it is seen that there are studies that emphasize that there is no relationship between gender and internalized stigma, as well as studies that found that internalized stigma is higher in men [23-25]. The significant decrease in stereotype endorsement and social withdrawal with increasing age may be the result of better adaptation to social environments as a result of good prognostic processes as well as insight and acceptance of the illness over the years. The results of a research study conducted on bipolar disorder patients in North India are similar to our findings [26].

The reasons for the internalization of stigmatization by society in mental illnesses and the related factors are not clear in the literature. However, our research provides insight into the relationship between stigma and religious coping. When both our research and the literature data are synthesized, it makes us think that the effect of negative and positive religious coping attitudes on mental states in individuals with religious beliefs is a remarkable issue. A review (2006) of 850 studies on the relationship between religiosity and mental health revealed that religious attitudes are associated with life satisfaction, lower stress levels, lower suicide risk, and a better psychological state [27]. However, positive and negative religious attitudes can vary according to society. Differences in belief systems make it difficult to reveal the relationship between mental health and religion. The fact that negative religious coping and internalized stigma sub-dimensions were associated in our study suggests that religious attitudes should be examined in detail amongst bipolar patients. It may be helpful to consider religious attitudes and religious coping methods in interviews with patients and in combating stigma.

Our study was notable in that it was conducted on a special group of patients such as bipolar disorder and evaluated both self-stigmatization and religious coping together. In addition, it offers a perspective on the evaluation of both psychosocial interventions in the patient group with mental disorders. The limitations of our study are that it was a cross-sectional study conducted only on bipolar disorder patients, it could not address other factors that could affect self-stigma and religious coping strategies, and it was conducted only on a Muslim patient population. Another limitation of our study is the lack of data on the age of onset of the disease, the duration of the disease, and compliance with treatment. Since internalized stigma may be higher in the early stages of the disease than in a chronic patient, it was not possible to evaluate the change over time.

## Conclusions

The results of our study tested religious coping as a factor that might be associated with internalized stigma in bipolar disorder patients and revealed significant relationships in some sub-dimensions of stigma. Since stigma is an important stressor in mental illnesses, individuals can activate some coping attitudes against this difficulty. Religious coping is a frequently used mechanism in individuals with a religious belief system, and the relationship between religious coping and internal stigma in bipolar mood disorder has not been explored before in the literature. The findings of our study brought to mind that stigma and religious dating attitudes may have a two-way interaction. Although positive or negative religious coping attitudes are thought to be an issue that needs to be addressed in the fight against internalized stigma in bipolar disorder, further clinical research is needed in this area.
